# One Disease, Multiple Hits: Acute Myeloid Leukemia With t(10;11) Presenting With Leukemic Lung Infiltration as First Presentation and Facial Nerve Palsy at Relapse

**DOI:** 10.7759/cureus.43726

**Published:** 2023-08-18

**Authors:** Thuraya AL-Busaidi, Nawaf AL-Muqaimi, Fatma Al-Bulushi

**Affiliations:** 1 Hematology, Sultan Qaboos University Hospital, Muscat, OMN; 2 Surgery, Sultan Qaboos University Hospital, Muscat, OMN

**Keywords:** monoblasts, mll, facial nerve palsy, leukemic lung infiltration, aml

## Abstract

Acute myeloid leukemia (AML) with t(10;11) is associated with poor outcomes. We report a rare case of monoblastic AML with mixed lineage leukemia (MLL) gene rearrangement t(10;11)(p11.2;q23) in a patient with extensive leukemic lung infiltration on his initial presentation leading to rapid deterioration following induction chemotherapy. Complete remission with negative cytogenetics was achieved following a limited induction due to acute respiratory failure. The patient subsequently relapsed with central nervous system involvement presenting with unliteral left lower motor neuron facial nerve palsy with cerebrospinal fluid positive for monoblasts.

Few unusual and challenging features were encountered with this patient including leukemic lung infiltration with extremely high lactate dehydrogenase (LDH) at the time of his initial presentation, rapid onset acute respiratory failure with no other identified causes within 48 hours of commencing induction chemotherapy. Additionally, achieving remission with only two days on induction chemotherapy and finally a stormy relapse with central nervous system involvement and left facial nerve palsy.

## Introduction

Chromosomal translocations involving mixed lineage leukemia (MLL) gene represent frequent cytogenetic abnormalities found in hematologic malignancies, occurring in 5-6% of patients with acute myeloid leukemia [1]. t(10;11) is rare with an estimated frequency of 0.3% of MLL rearranged acute leukemia cases [2]. This recurrent genetic abnormality is associated with a distinct disease entity characterized by high early morbidity and mortality, as well as high rates of relapse [3]. Additionally, it's associated with poor overall survival and poor event-free survival [3,4]. 

Extramedullary leukemic involvement in acute myeloid leukemia (AML) is infrequent, with an estimated incidence of approximately 11% based on retrospective data [5]. Among the affected sites, skin is the most common location, accounting for a significant proportion. Central nervous system (CNS) is involved in approximately 23% of cases of extramedullary leukemic involvement, while the pleura is affected in around 7% of cases [5]. Leukemic lung infiltration is more commonly observed in relapsed and refractory settings of AML, while it is rarely seen at the initial presentation of the disease [6].Monoblastic AML in general has high tendency for extramedullary infiltration. Distinctively MLL rearranged AML is associated with high tendency for extramurally involvement [5]. Additionally symptomatic facial nerve involvement in AML patients is rare but has been reported in the literature [7].

## Case presentation

A previously healthy 23-year-old male presented in April 2023 with a two-week history of fever, drenching night sweats and extreme fatigue. He was diagnosed with monoblastic AML based on a bone marrow biopsy with estimated blasts of 92%. The bone marrow aspirate revealed packed marrow with medium- to large-size blasts, no Auer rods seen. Based on flow cytometry these blasts are CD34+ (heterogneous and dim), CD117+, CD13+ (heterogenous), CD33+, CD4+ (aberrant), CD14+ (heterogenous), CD64+, HLA-DR+, CD11c+, MPO+ (dim) and CD56+. The blasts are negative for B and the rest of T cell markers. Cytogenetics showed MLL rearrangement t(10;11)(p11.2;q23) in addition to ring 13 chromosome. It's worth noting that our patient did not exhibit any features associated with ring chromosome syndrome. 

**Figure 1:**
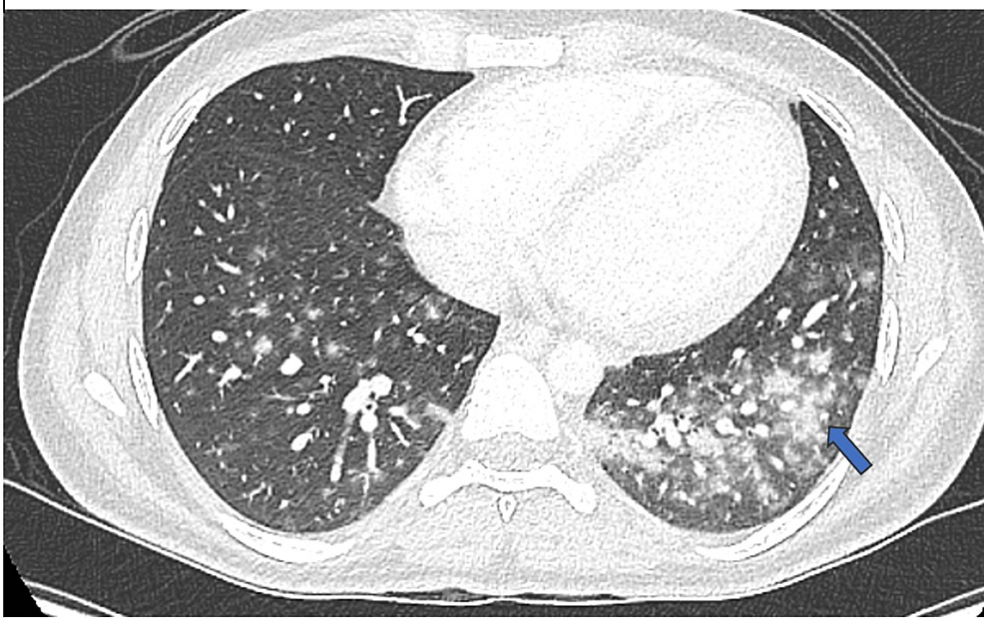
High-resolution computed tomography (HRCT) of the lungs showing large clusters of centrilobular nodules noted within both lower lobes.

The patient also had evidence of spontaneous tumor lysis syndrome (TLS) on admission with creatinine 118 umol/L phosphate 1.86 mmol/L, uric acid 0.45 mmol/L, lactate dehydrogenase (LDH) was 6163 U/L on initial presentation. WBC 79x109/L, platelets 109x109/L, absolute neutrophil count (ANC) 1.1x109/L. Aggressive TLS management measures were taken with allopurinol and hydration. Rasburicase could not be administered due to G6PD deficiency. Cytoreduction therapy with hydroxyurea was initiated prior to starting induction therapy with cytarabine 100 mg/m2 and daunorubicin 60 mg/m2. Of note, WBC came down to 10.3x109/L prior to commending induction chemotherapy and LDH was down to 1800 U/L on the D+1 of induction. Additionally, the patient had persistent high-grade fever up to 39.5 since his admission. Septic workup was all negative. CT chest revealed small mediastinal adenopathy, and extensive bilateral lower lobes nodule (Figure 1). He underwent bronchoscopy on D+2 of induction to assess for infective causes including invasive fungal infection. Bronchoscopy was normal, and bronchoalveolar lavage (BAL) samples were taken. 

All cultures from BAL came back negative. Patient remained well from a respiratory perspective despite the notable findings observed in the high-resolution computed tomography (HRCT) upon admission, maintaining this status until D+3 of the induction. 

**Figure 2:**
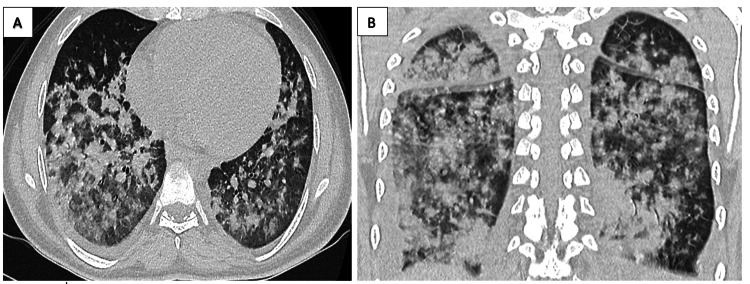
Worsening bilateral extensive lung nodules 48 hours from induction chemotherapy.

On D+3 of induction he developed acute respiratory distress and significant desaturation on room air. Chest x-ray showed diffuse bilateral opacities more, which was further confirmed with CT chest (Figure 2). His WBC on D+3 of induction was 0.8x109/L, ANC 0.4x109/L and the LDH level rose again, reaching 5721 U/L.Within less than 12 hours, his work of breathing became worse, with propound hypoxia and increased work of breathing. Chemotherapy was held and patient was admitted to intensive care unit as intubation was imminently needed. The patient remained on non-invasive ventilation (NIV) with signs of stabilization within 48 hours of admission to intensive care unit; thankfully he did not require intubation. Antimicrobial coverage was further optimized with tigecycline, colistin and meropenem in addition to voriconazole. Repeat bronchoscopy was not feasible as intubation will be inevitable in such circumstances. 

**Figure 3:**
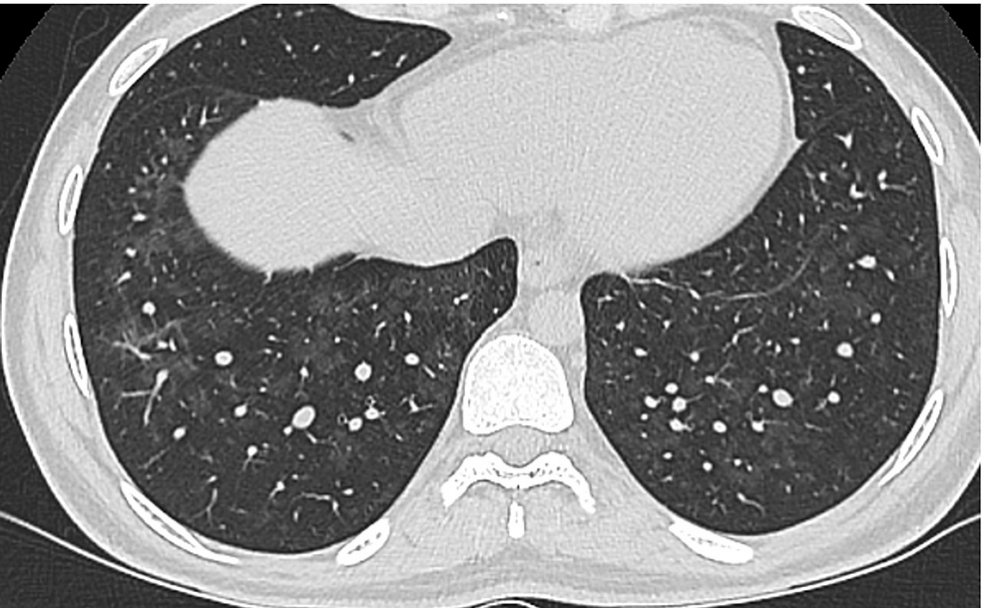
High-resolution computed tomography (HRCT) of the lungs showing complete resolution of bilateral extensive lung nodules two weeks following induction.

Chemotherapy remained on hold given acute respiratory distress however he recovered his counts robustly within 10 days. Repeat bone marrow biopsy done on D+14 of induction revealed 1.4% blasts which was also confirmed by multiparameter flow cytometry. Cytogenetics also showed completed cytogenic remission. Upon count recovery he had robust thrombocytosis with platelet count approaching 1300. Myeloproliferative panel (MPN) came back negative. Lumbar puncture (LP) was done to assess for CNS involvement and was unremarkable. Repeat HRCT two weeks later showed complete resolution of lung infiltrates (Figure 3). 

Once cytogenetics and morphological complete remission (CR) were confirmed he moved on to receive his first consolation with high-dose cytarabine (HIDAC). A few days following count recovery he developed sudden onset left facial numbness and weakness, inability to close his eye or mouth, absent forehead wrinkles. Clinical examination was consistent with left facial nerve palsy. CT stroke protocol did not reveal any abnormality and MRI brain was negative for leptomeningeal involvement. Dedicated images of the mastoid bone to assess for compressive masses were also unremarkable. 

**Figure 4:**
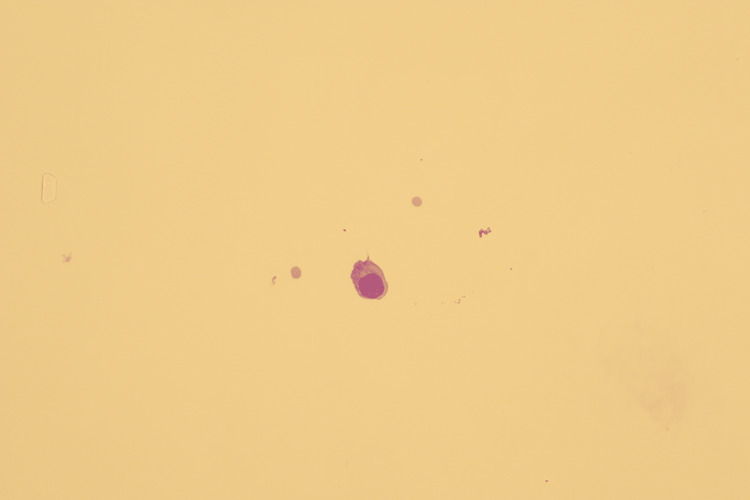
Monoblasts in the cerebrospinal fluid

LP was done which conformed presence of monoblasts in keeping with CNS relapse (Figure 4). Very rapidly within two days of this development, circulating blasts were noted in peripheral blood. Bone marrow biopsy confirmed relapsed disease with 31% blasts in the bone marrow. 

Following our patient’s early relapse with confirmed CNS involvement he was started on salvage chemotherapy azacitidine and venetoclax and continued on triple IT chemotherapy (methotrexate, cytosine arabinoside and hydrocortisone) twice weekly to address CNS disease. Our patient did not develop any cytopenia or required any transfusion support throughout the period of his first cycle of azacitidine and venetoclax. A bone marrow biopsy was done on D+28 which unfortunately revealed significant disease progression to fully packed marrow. Within a few days his white cell count was doubling approaching 30x109/L, additionally LDH was rising steadily to 6500 U/L within three days. He subsequently was started on salvage chemotherapy with FLAG-IDA (Fludarabine, Cytarabine, Idarubicin and G-CSF) and 14 days of venetoclax. He is planned for consolidative allogenic stem cell transplantation once second remission (CR2) is achieved. 

## Discussion

We present a case study highlighting several unusual presentations in a patient diagnosed with monoblastic AML with MLL rearrangement t(10;11)(p11.2;q23). These manifestations can be attributed to the inherent nature of the disease. The case exemplifies the aggressive behavior of this distinct disease evident at various levels, including the involvement of multiple extramedullary sites and an exceptionally early relapse. Furthermore, the patient experienced significant morbidity during the induction chemotherapy phase, further emphasizing the challenges associated with managing this aggressive form of acute myeloid leukemia. 

The first challenge encountered with this patient is a rapid onset respiratory failure following induction chemotherapy. This has been documented in the medical literature. Notably, Tryka et al. reported their observations of five patients with myeloblastic leukemia who experienced acute respiratory distress within four days of reaching the nadir in their cell counts [8]. This phenomenon, known as leukemic cell lysis pneumopathy, was initially described by Myers et al. in 1983 [9]. Histological analysis of tissue specimens obtained from four patients in the Myers et al. study revealed congestion of pulmonary arterioles, capillaries, and venules, along with the presence of leukocytes and blasts, small infarctions, and perivascular hemorrhage. This was attributed to pulmonary leukostasis. However, In 1992, Dombret et al. provided a significant insight into this phenomenon observed in the Myers et al. report [10]. Their patients, despite having markedly elevated white cell counts, did not display leukostasis-related symptoms. However, these symptoms rapidly emerged following the initiation of cytotoxic chemotherapy, accompanied by acute respiratory failure. The authors attributed the pulmonary injury to the lysis of leukemic cells in the lungs. Our patient's case lends further support to their theory. Notably, he had very low white cell count at the time of acute respiratory failure onset and his LDH increased dramatically within 24 hours, which could be indicative of cell lysis. Drawing from both our observations and Dombret et al. and Myers et al. studies, it is reasonable to suspect that the initial findings observed in the admission HRCT were indicative of leukemic lung infiltration. Subsequent to the commencement of cytotoxic chemotherapy, the patient's condition deteriorated due to cell lysis within the lung parenchyma, leading to acute respiratory distress.

Post-mortem examinations of the lungs of patients with AML have detected leukemic cell infiltration within the pulmonary parenchyma in a significant proportion of patients, ranging from 31% to 66% [11]. The presence of these pulmonary infiltrates is associated with a rapid deterioration in the patient's clinical condition due to acute respiratory failure.

Diagnosing leukemic cell lysis pneumopathy can be challenging given other differentials that need to be excluded in this unique population particularly infectious causes and leukostasis more importantly in cases where the white cell count is extremely elevated. The pattern of involvement and timeline of lung involvement are also important factors to be considered to aid the diagnosis and best next diagnostic approach. Based on Tenholder and Hooper's retrospective review of 139 adults with leukemia and pulmonary infiltrates, local disease during treatment is likely to be infectious compared to diffuse lung involvement which is more likely to be noninfectious. In addition, parenchymal lung infiltration appearing before commencing chemotherapy or within 72 hours of initiating treatment is unlikely to be infectious [12]. Additionally, true leukemic lung infiltrates are usually of interstitial pattern on HRCT. This pattern is formed by blasts aggregates around smaller bronchi and blood vessels which form nodules [13]. This description is consistent with our patient HRCT findings. Our patient presented with evidence of spontaneous tumor lysis syndrome, which resolved rapidly following appropriate measures and effective cytoreduction prior to initiating chemotherapy. Interestingly, despite the presence of lung infiltration upon admission, the patient did not display any clinical signs of leukostasis and remained well, without respiratory distress.

However, a rapid deterioration in the patient's condition occurred within 48 hours of starting cytotoxic chemotherapy. Additionally, this deterioration occurred after cytoreduction and following initiation of induction chemotherapy. These facts combined are not in keeping with leukostasis pattern where signs and symptoms of respiratory distress if any are more expected at the time when he had significant leukocytosis not at the time when his white cell count reached the nadir. However, it's important to acknowledge that the possibility of leukostasis in this case cannot be completely dismissed. 

Although we were unable to confirm the diagnosis through pathological confirmation, several factors support the presence of leukemic lung infiltration in our patient. These include findings from CT chest images demonstrating a characteristic pattern of lung involvement, exclusion of infectious causes, markedly elevated levels of LDH, rapid deterioration with acute respiratory failure following induction, and complete resolution of lung nodules upon achieving remission. Collectively, these findings strongly support the likelihood of leukemic lung infiltration in our patient.

Early recognition of this potential complication is essential prior to commencing induction chemotherapy as it poses a major therapeutic dilemma. Our patient's deterioration led to interruption of induction chemotherapy. Aggressive supportive measures including higher transfusion targets were instituted during this period to mitigate the risk of alveolar hemorrhage and worsening respiratory status during the period of expected thrombocytopenia. Despite achieving adequate cytoreduction, this complication remained unavoidable. Nevertheless, early recognition plays a vital role in implementing the most appropriate supportive care and broadening the scope of the differential diagnosis beyond infectious and leukostasis.

The second distinct presentation in the same patient is unilateral left facial nerve palsy with no evidence of compressive mass on images. Most of the reported cases of isolated facial nerve palsy in AML and acute lymphoid leukemia (ALL) are in the context of nerve compression due to mastoiditis, however myeloblasts can directly infiltrate the nerves in the absence of compressive masses [7]. It's also important to note that MRI not necessarily can delineate direct nerve infiltration. Our patient has positive cerebrospinal fluid (CSF) for monoblasts. We did not identify any evidence of infection including mastoiditis however we empirically treated him for herpes simplex virus (HSV) infection with therapeutic valacyclovir for a total of seven days followed by prophylaxis which he was receiving regularly. 

It is important to consider direct nerve infiltration by leukemic cells as a potential cause of isolated facial nerve palsy in AML, even in the absence of identifiable compressive masses. While imaging may not always provide definitive evidence, CSF analysis can help confirm the presence of leukemic involvement in such cases [14]. 

During our literature search, we came across two reported cases with a similar pattern of presentation to our patient. These cases were documented by the MD Anderson group in 2015 and involved patients with t(10;11) translocation [3]. Like our patient, these cases experienced rapid onset respiratory failure and tumor lysis syndrome during induction chemotherapy. However, in contrast to our patient, there was no evidence of CNS involvement in those reported cases.

## Conclusions

Monoblastic AML with MLL rearrangement t(10;11)(p11.2;q23) is a distinct entity with aggressive nature. Vigilant assessment and early recognition of potential lung involvement are essential during early days of induction chemotherapy as rapid deterioration in clinical status particularly acute respiratory failure can be devastating. Higher mortality and morbidity rates during induction chemotherapy are described due to disseminated intravascular coagulation (DIC), acute respiratory failure and tumor lysis syndrome. Early consolidation with allogenic stem cell transplantation when feasible should be prioritized.
